# Evaluation and estimation of compressive strength of concrete masonry prism using gradient boosting algorithm

**DOI:** 10.1371/journal.pone.0297364

**Published:** 2024-03-05

**Authors:** Lanh Si Ho, Van Quan Tran

**Affiliations:** 1 University of Transport Technology, Thanh Xuan, Hanoi, Vietnam; 2 Civil and Environmental Engineering Program, Graduate School of Advanced Science and Engineering, Hiroshima University, Hiroshima, Japan; Universiti Teknologi Malaysia, MALAYSIA

## Abstract

The compressive strength (CS) of the hollow concrete masonry prism is known as an important parameter for designing masonry structures. In general, the CS is determined using laboratory tests, however, laboratory tests are time-consuming and high-cost. Thus, it is necessary to evaluate and estimate the CS using different methods, for example, machine learning techniques. This study employed Gradient Boosting (GB) to evaluate and predict the CS of hollow masonry prism. The database consists of 102 hollow concrete specimens taken from different previous published literature used for modeling. The output is the CS of the hollow masonry prism, while the inputs include the compressive strength of mortar (f_m_), the compressive strength of blocks (f_b_), height-to-thickness ratio (h/t), the ratio of f_m_/f_b_. To reduce the overfitting problem, this study used K-Fold cross-validation, then particle swarm optimization (PSO) was employed to obtain the optimum hyperparameter. The GB model then was modeled using the optimum hyperparameters. The results showed that the GB model performed very well in evaluating and predicting the CS of the hollow masonry prims with a high prediction accuracy, the values of R^2^, RMSE, MAE, and MAPE are 0.977, 0.803 MPa, 0.612 MPa, and 0.036%, respectively. The performance of the GB model in this study outperformed in comparison to six different machine learning models (decision tree, linear regression, random forest regression, ridge regression, Artificial Neural network, and Extreme Gradient Boosting) used in previous studies. The results of sensitivity analysis using SHAP and PDP-2D indicate that the CS is strongly dependent on the f_b_ (with a mean SHAP value of 3.2), h/t (with a mean SHAP value of 1.63), while the f_m_/f_b_ (with a mean SHAP value of 0.57) had a small effect on the CS. Thus, it can be stated that this research provides a good method to evaluate and predict the CS of the hollow masonry prism, which can bring good knowledge for practical application in this field.

## 1. Introduction

The compressive strength (CS) of hollow concrete masonry is known as one of the most vital mechanical factors, which strongly affects the technical and economical aspects of the masonry structures [1, 2]. The CS is known as one of the main mechanical factors that remarkably affect the safety and economy of the structures [3]. However, it is quite challenging to determine the CS due to the complex components of masonry structures [4, 5]. For example, masonry structures include unreinforced and reinforced hollow interlocking compressed stabilized earth, braced frames incorporating masonry infills, steel reinforced grout composites and masonry substrate, and masonry concrete structures [6–8]. Many previous theoretical studies have investigated the strength and behavior of masonry hollow prisms [9–11]. Besides, some analytical models have been developed on the basis of equilibrium and deformation compatibility equations to estimate the CS. It was reported that the CS can be estimated using Eurocode 6 [12], which considers the compressive strength of each component material such as block and mortar, etc. Besides, many previous studies used empirical models to estimate the CS based on the results from the laboratory [13–19].

Most previous studies mainly considered the compressive strength of masonry unit (f_b_) and compressive strength of mortar (f_m_), however, the CS of hollow blocks is also affected by some factors such as the dimension of the prism and mortar [15]. To consider other factors, the previous study established the empirical model to predict the CS using VF_b_ (volume fraction of masonry unit), H_p_/B (height of masonry prism/width of the masonry unit), and VR_mH_ (volume ratio of bed joints to mortar) as the input variables [15]. The results of the previous study indicated that the proposed models performed well in predicting the CS with a determination coefficient R^2^ = 0.88. Nevertheless, this previous study was only applied to a specific masonry unit, the dimension of the masonry unit, and the thickness of the mortar joint. Furthermore, it was indicated that it is difficult to use the empirical model to estimate the CS with complex variables [20].

Recently, machine learning (ML) techniques have been applied popularly in estimating problems in civil engineering, particularly in concrete [21–25]. Besides, different machine learning techniques such as Gene Expression Programming (GEP), Adaptive Neuro-Fuzzy Inference System (ANFIS), and multiple linear regression (MLR) to estimate the compressive, flexural strength, and maximum deflection of concrete and reinforced concrete panels [26–28]. ML techniques are known as good solutions to predict the properties of materials because they can deal with problems having multiple variables. Although many previous studies used ML techniques to forecast the compressive strength of concrete, only few studies have been implemented to estimate the CS of the hollow concrete masonry prism using ML techniques. A previous study used artificial neural networks (ANN) and adaptive neuro-fuzzy inference systems (ANFIS) to estimate the CS of hollow concrete masonry prisms. The previous study used three main variables as the input parameters, including the height-to-thickness ratio, mortar compressive strength, and unit compressive strength. The results of the previous study indicated that the proposed models gave a high prediction accuracy with small error rates [29].

Previous studies used empirical models to predict compressive strength, and it was stated that those empirical models strongly depended on the height-to-thickness ratio of prisms, mortar compressive strength, and compressive strength of blocks. Nevertheless, these empirical models need to be re-assessed when new test results are updated [29]. A previous study developed a cellular automated model to estimate the cracking patterns of vertically loaded masonry wallets [30]. Garzón-Roca et al. used ANN and fuzzy logic models to predict the compressive strength of brick masonry [31, 32]. Besides, the ANN model was used to estimate the masonry failure surface subjected to biaxial compressive stress [33]. Asteris et al. [34] used a backpropagation artificial neural network to estimate the compressive strength of brick masonry, and the results indicated that the proposed models fitted well with the experimental result.

Some previous studies used ML techniques namely fuzzy set, fuzzy logic, and neural network to predict the compressive strength of masonry hollow blocks [35,36]. The data used for modeling was attained from the experimental works, including both direct tests and non-destructive tests (rebound hammer and ultrasonic pulse velocity) in order to estimate the compressive strength of masonry. The results of the sensitivity analysis of the previous study revealed that the masonry unit’s compressive strength is the most influential factor affecting the masonry compressive strength [37]. Besides, Fakharian et al. [38] also indicated that the most vital parameter affecting the CS prediction of hollow concrete prisms is the compressive strength of concrete blocks. The results show that the ANN model achieved the best performance in predicting the CS with the value of R = 0.950 and the value of 6.92%. The prediction of the CS using ANN and ANFIS was conducted with 66 datasets obtained from literature, in which the input variables include f_m,_ f_b,_ and H_p_/B [39]. The results indicated that the ANFIS performed well in predicting the CS with a mean ratio between the actual value to the estimated value of 0.983. Furthermore, a previous study employed experimental datasets and used different ML techniques such as ANN, random forest regression, and XGBoost to forecast the CS. In that study, in addition to f_b_, f_m_, other input variables such as the dimension of masonry and unit material were also considered. The results showed that ANN models achieved the highest prediction accuracy with R^2^ = 0.95 and RMSE = 1.83 MPa. However, almost previous studies have not completed evaluating the influence of each variable on the CS prediction.

Gradient boosting (GB) is based on the boosting technique, which is considered as an ensemble technique proposed by Friedman [40]. The GB algorithm is built by incorporating weak learners into better learners via an iterative sequence [41]. As a result, the performance of the GB algorithm could be strengthened, leading to a reduction in the total error and the loss of the model [42]. Previous studies stated that the GB technique has a basic advantage in preventing overfitting problems and this technique uses fewer computational resources [42,43]. The GB algorithm has been employed in many studies for predicting the compressive strength of concrete [25,44]. A previous study was conducted to compare the prediction performance between GB, SVM, and random forest models in predicting genomic breeding values, and it was concluded that the GB algorithm outperformed other algorithms [45]. Six different machine learning techniques, consisting of GB were used to estimate the stability of the open stop-hanging wall, it was concluded that among the six ML techniques, the GB technique outperformed than other ones [46]. The GB technique was also used to estimate the structure damage caused by blasting vibration, and it was found that the GB model obtained a high accuracy in predicting the structure damage [47].

Based on the above literature and the outstanding performance of the GB algorithm, this research aims to employ the Gradient Boosting (GB) technique to predict and evaluate the CS of the hollow concrete masonry prism. As aforementioned, previous studies used different models to estimate the CS, however, the results showed a low prediction accuracy. Therefore, to improve the high accuracy of the proposed model, the K-Fold Cross-Validation and the hyper-parameters tuning process were conducted by Particle Swarm Optimization (PSO) in this study. Furthermore, the influence of each input variable as well as coupled input parameters on the compressive strength was evaluated using sensitivity analysis via Partial dependence plots (PDP-2D) and Shapley Additive exPlanations (SHAP).

## 2. Research significance

The performance of hollow concrete masonry blocks is generally governed by compressive strength (CS). The CS is known as the most important parameter of masonry structures. In general, the CS of hollow concrete masonry blocks is determined by the experiment test in the laboratory, which consumes time and cost. Besides, due to the complex components of masonry structures, the determination of the CS is a challenging task. Thus, many theoretical and empirical approaches have been developed to estimate the CS, but it was indicated that it is difficult to use the empirical model to predict the CS with complex variables. Therefore, recently there have been several studies using machine learning (ML) techniques to forecast the CS of the hollow concrete masonry block. Nevertheless, previous studies did not have a high prediction accuracy. Based on those remaining issues in previous studies, this study used the GB model, which is known as a basic advantage in preventing overfitting problems. Furthermore, to enhance the prediction accuracy of the model, the K-Fold cross-validation with 10 iterations was applied. Besides, optimum hyper-parameters for the proposed model were obtained using the PSO. The study highlights the significance of different parameters that directly influence the compressive strength of the hollow concrete masonry block. The outcome of this study is to provide actionable knowledge, which could support the design of the engineer in the practical application of hollow concrete masonry prisms.

## 3. Data collection and description of the database

**Fig 1:**
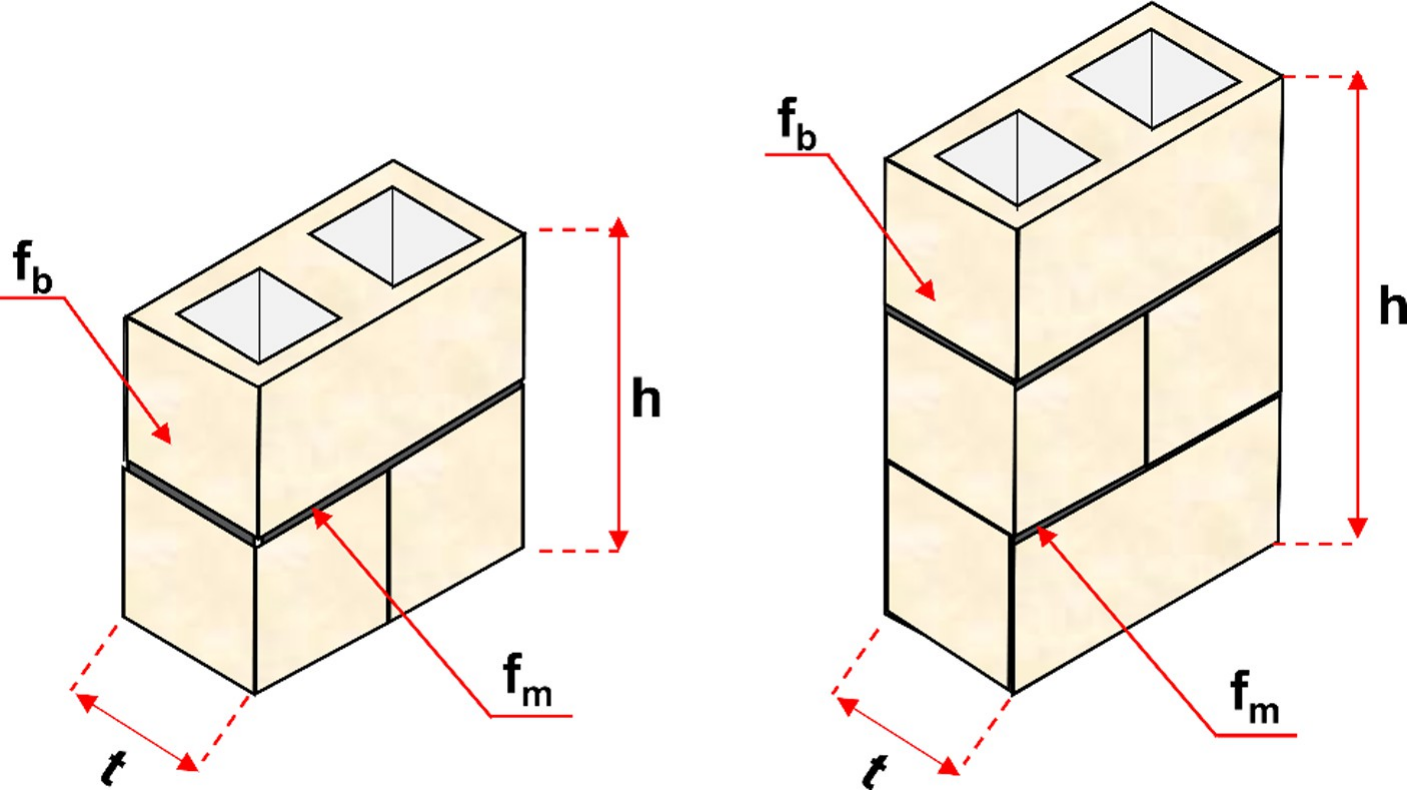
The geometry and input variables influence the CS of the hollow concrete prisms.

In this study, 102 hollow concrete specimens were derived from the literature [29,44,48–55]. The database includes three input variables, including the compressive strength of mortar (f_m_), the compressive strength of blocks (f_b_), height-to-thickness ratio (h/t), the ratio of f_m_/f_b_ and the output is the compressive strength of prisms (CS). The geometry and input variables influencing the CS of the hollow concrete are shown in **Fig 1**. From Table 1, it can be seen that the values of f_m_ range from 4.6 to 22.8 MPa, while the f_b_ values vary from 15.4 to 40.5 MPa. The h/t and f_m_/f_b_ vary from 1.8 to 4.3 and from 0.19 to 1.48, respectively. The values of output CS are in the range of 11.3 to 27 MPa. The details of input variables and output can be found in Table 1.

**Table 1:** Experimental data used for modeling in this study.

No.	f_m_ (MPa)	f_b_ (MPa)	h/t	f_m_/f_b_	CS (MPa)
**1**	17.50	27.20	2.80	0.64	24.67
**2**	17.50	40.50	2.80	0.43	27.00
**3**	17.50	24.60	2.80	0.71	20.75
**4**	10.40	24.60	2.80	0.42	18.20
**5**	4.60	24.60	2.80	0.19	17.70
**6**	17.50	23.00	4.30	0.76	21.15
**7**	17.50	15.90	3.90	1.10	14.90
**8**	17.50	21.20	3.90	0.83	16.75
**9**	17.50	18.10	1.80	0.97	14.25
**10**	22.80	17.20	4.20	1.33	13.93
**11**	14.80	17.20	4.20	0.86	13.80
**12**	22.80	26.10	4.20	0.87	20.69
**13**	14.80	26.10	4.20	0.57	19.79
**14**	22.80	15.40	2.70	1.48	11.66
**15**	14.80	15.40	2.70	0.96	11.31
**16**	22.80	23.80	2.70	0.96	18.82
**17**	14.80	23.80	2.70	0.62	20.76
**18**	22.80	15.60	2.00	1.46	13.45
**19**	14.80	15.60	2.00	0.95	14.82
**20**	22.80	27.70	2.00	0.82	22.00
**21**	14.80	27.70	2.00	0.53	22.69
**22**	15.10	19.70	4.20	0.77	15.80
**23**	16.70	19.70	4.20	0.85	16.00
**24**	17.20	19.70	4.20	0.87	15.93
**25**	5.70	19.70	4.20	0.29	15.38
**26**	14.70	19.70	4.20	0.75	16.40
**27**	18.20	19.70	4.20	0.92	16.28
**28**	14.20	32.20	4.20	0.44	25.41
**29**	14.20	22.00	4.20	0.65	18.00
**30**	14.20	21.30	4.20	0.67	19.38
**31**	14.20	20.20	4.20	0.70	17.86
**32**	14.20	20.00	4.20	0.71	16.14
**33**	14.20	15.60	4.20	0.91	12.76
**34**	15.40	20.00	3.10	0.77	17.40
**35**	20.20	35.70	3.10	0.57	24.20
**36**	9.20	20.00	3.10	0.46	17.80
**37**	21.20	20.00	3.10	1.06	21.25
**38**	15.40	25.70	3.10	0.60	20.60
**39**	26.50	25.70	3.10	1.03	25.50
**40**	21.20	20.00	2.10	1.06	24.90
**41**	26.50	20.00	3.10	1.33	21.40
**42**	21.20	25.70	2.10	0.82	26.00
**43**	15.60	19.80	2.90	0.79	13.90
**44**	12.20	19.80	2.90	0.62	13.22
**45**	5.00	19.80	2.90	0.25	11.89
**46**	4.30	19.80	2.90	0.22	10.82
**47**	15.60	17.60	2.90	0.89	13.76
**48**	12.30	17.60	2.90	0.70	11.14
**49**	5.00	17.60	2.90	0.28	10.14
**50**	4.30	17.60	2.90	0.24	9.75
**51**	12.20	13.50	2.90	0.90	9.10
**52**	5.00	13.50	2.90	0.37	8.74
**53**	4.30	13.50	2.90	0.32	8.39
**54**	5.00	10.90	2.90	0.46	7.24
**55**	4.30	10.90	2.90	0.39	6.63
**56**	4.90	9.00	4.20	0.54	7.11
**57**	4.90	13.40	4.20	0.37	9.20
**58**	9.10	14.10	2.00	0.65	17.00
**59**	14.00	14.10	2.00	0.99	15.80
**60**	9.10	20.8	2.00	0.44	23.00
**61**	9.10	17.70	2.00	0.51	18.60
**62**	9.10	23.30	2.00	0.39	20.50
**63**	9.10	28.30	2.00	0.32	23.50
**64**	9.10	31.10	2.00	0.29	23.80
**65**	9.10	35.00	2.00	0.26	23.70
**66**	9.10	38.00	2.00	0.24	29.20
**67**	4.60	14.10	2.00	0.33	15.20
**68**	4.60	17.70	2.00	0.26	17.50
**69**	4.60	20.80	2.00	0.22	20.60
**70**	4.60	23.30	2.00	0.20	17.30
**71**	4.60	28.30	2.00	0.16	22.30
**72**	4.60	31.10	2.00	0.15	21.00
**73**	4.60	35.00	2.00	0.13	21.50
**74**	4.60	38.00	2.00	0.12	24.40
**75**	10.80	14.10	2.00	0.77	14.70
**76**	10.80	23.30	2.00	0.46	20.80
**77**	10.80	38.00	2.00	0.28	27.50
**78**	3.90	14.10	2.00	0.28	13.70
**79**	3.90	23.30	2.00	0.17	15.60
**80**	3.90	38.00	2.00	0.10	24.20
**81**	14.00	23.30	2.00	0.60	20.90
**82**	14.00	38.00	2.00	0.37	31.00
**83**	6.70	14.10	2.00	0.48	15.40
**84**	6.70	23.30	2.00	0.29	17.20
**85**	6.70	38.00	2.00	0.18	28.10
**86**	22.80	35.10	3.00	0.65	24.20
**87**	22.80	35.10	2.00	0.65	25.70
**88**	14.90	25.90	2.10	0.58	19.24
**89**	10.50	13.90	3.10	0.76	11.74
**90**	10.50	13.90	3.10	0.76	10.08
**91**	5.60	23.15	3.10	0.24	11.66
**92**	5.60	36.75	3.10	0.15	18.24
**93**	10.49	23.15	3.10	0.45	13.86
**94**	10.49	36.75	3.10	0.29	20.27
**95**	13.73	23.15	3.10	0.59	16.22
**96**	13.73	36.75	3.10	0.37	27.03
**97**	5.60	23.15	5.20	0.24	10.15
**98**	5.60	36.75	5.20	0.15	15.84
**99**	10.49	23.15	5.20	0.45	11.12
**100**	10.49	36.75	5.20	0.29	17.56
**101**	13.73	23.15	5.20	0.59	14.43
**102**	13.73	36.75	5.20	0.37	25.35

**Fig 2:**
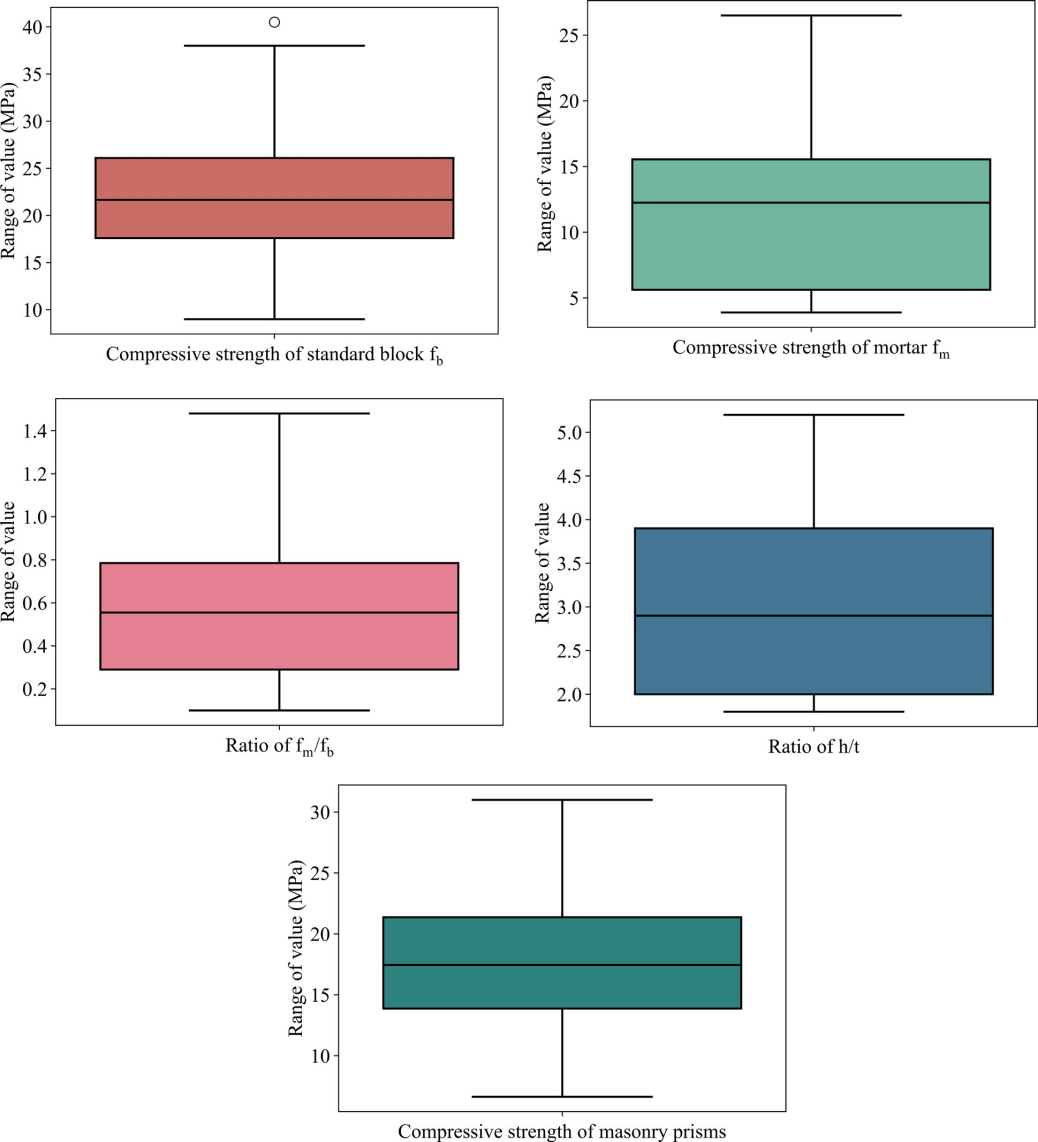
The magnitude of input variables and output.

The magnitude of input variables and output are shown in **Fig 2**. It can be observed that the compressive strength of standard block f_b_ has a medium value of 22 MPa with a range of deviation (from 10 to 39 MPa). The median value of the compressive strength of mortar f_m_ is approximately 13 MPa. The median values of f_m_/f_b_ and h/t are 0.58 and 2.9, respectively. The compressive strength of masonry prisms has a median value of 18 MPa.

**Fig 3:**
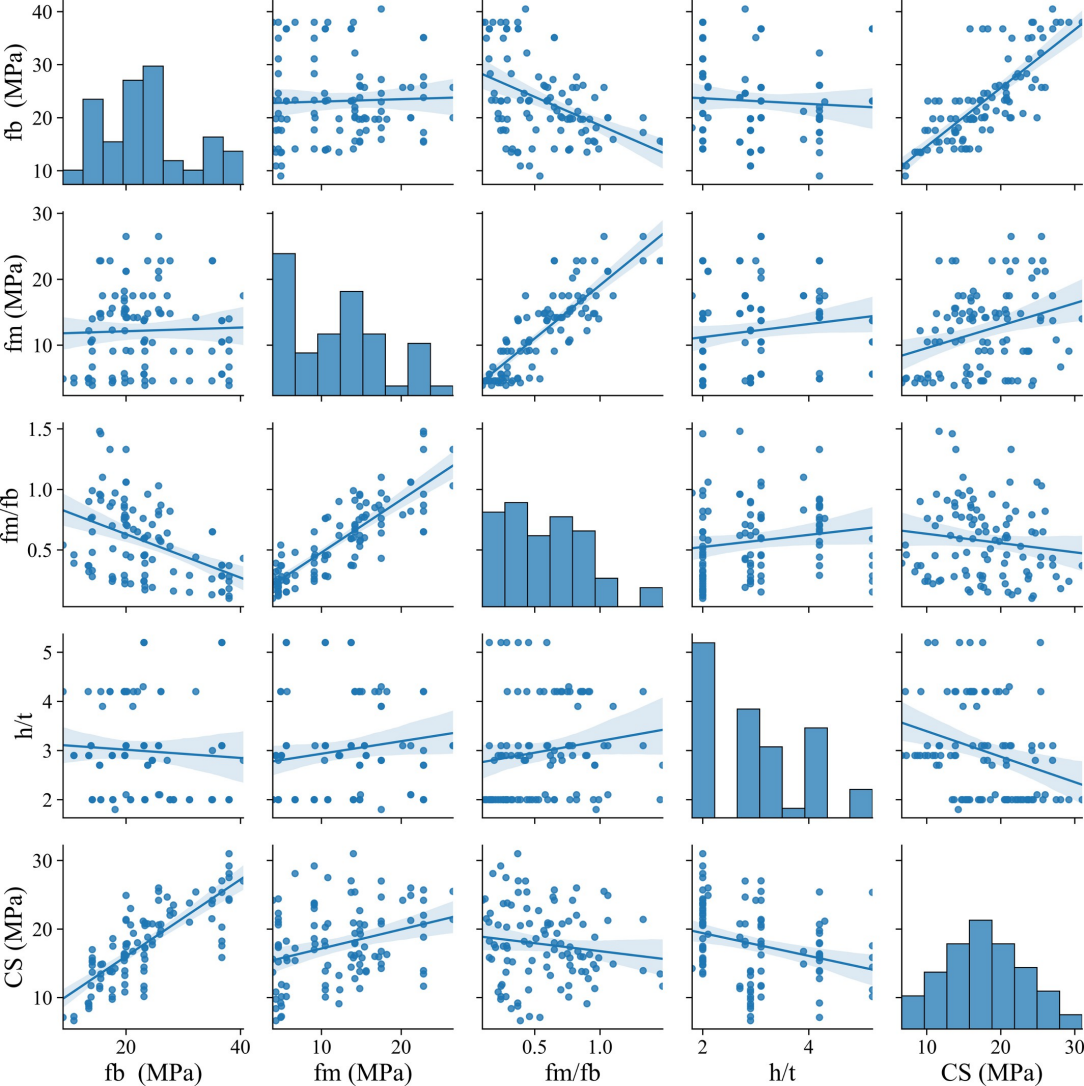
Distribution of data among input variables and output.

The data distribution among input variables and output are presented in **Fig 3**, containing f_b_, f_m_, f_m_/f_b_, h/t, and CS (output). The f_b_ values range from 10 to 40 MPa, and are mostly concentrated at 25 MPa. The f_b_ is mostly independent of f_m_ and h/t, while f_b_ has a negative relation with increasing f_m_/f_b_. There is a strong relationship between f_b_ and CS, the f_b_ increases linearly with increasing CS value. Besides, f_m_ increased linearly with f_m_/f_b_ values, and while there is a large variation between f_m_ and CS, f_m_ slightly increased with increasing CS values. f_m_/f_b_ ranges from 0 to 1.0 and is mostly located from 0.5 to 1.0. There is a large variation of f_m_/f_b_ with the change of h/t, the f_m_/f_b_ slightly increases with increasing in h/t. The f_m_/f_b_ also largely varies with CS, the f_m_/f_b_ slightly reduces with increasing in h/t. From Fig 3, it can be seen that the values of h/t vary from 2 to 5. h/t has a large scatter with the change of CS values. The values of h/t slightly reduce with increasing CS values. The values of CS have a range of 10 to 30 MPa and are mostly located around 18 MPa.

**Fig 4:**
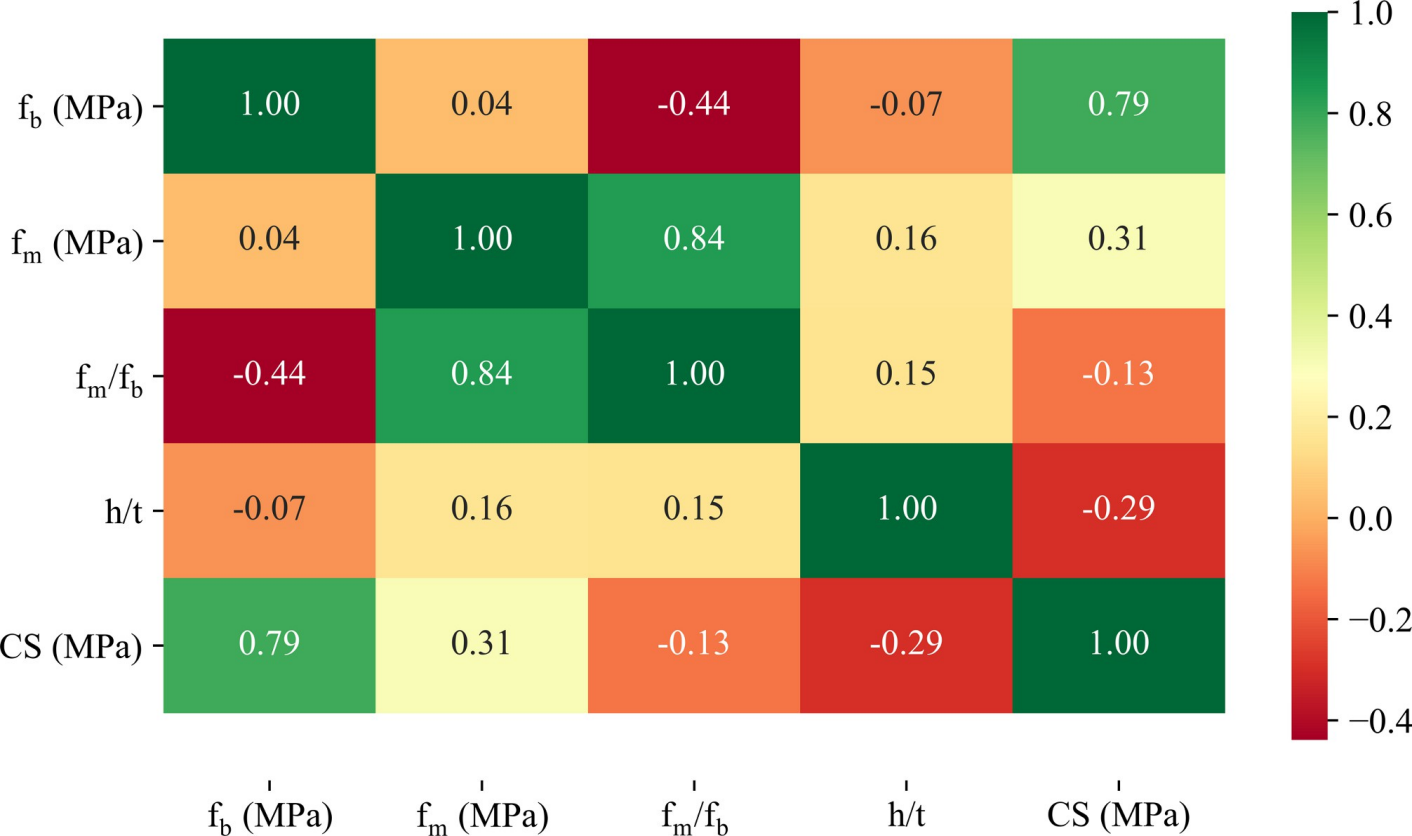
Correlation matrix of the input and output variables.

The correlation matrix between input and output variables is shown in **Fig 4**. Among the input variables, the greatest correlation (R = 0.84) was found between f_m_ and f_m_/f_b_, while the lowest was achieved between f_m_ and f_b_ (R = 0.04). The highest correlation between input and output was achieved for the CS and f_b_ (R = 0.79). From this result, it can be said that the relationships between input and output as well as among inputs are not high, thus, these variables should be considered in the modeling.

## 4. Machine learning methods

### 4.1. Gradient boosting (GB)

**Fig 5:**
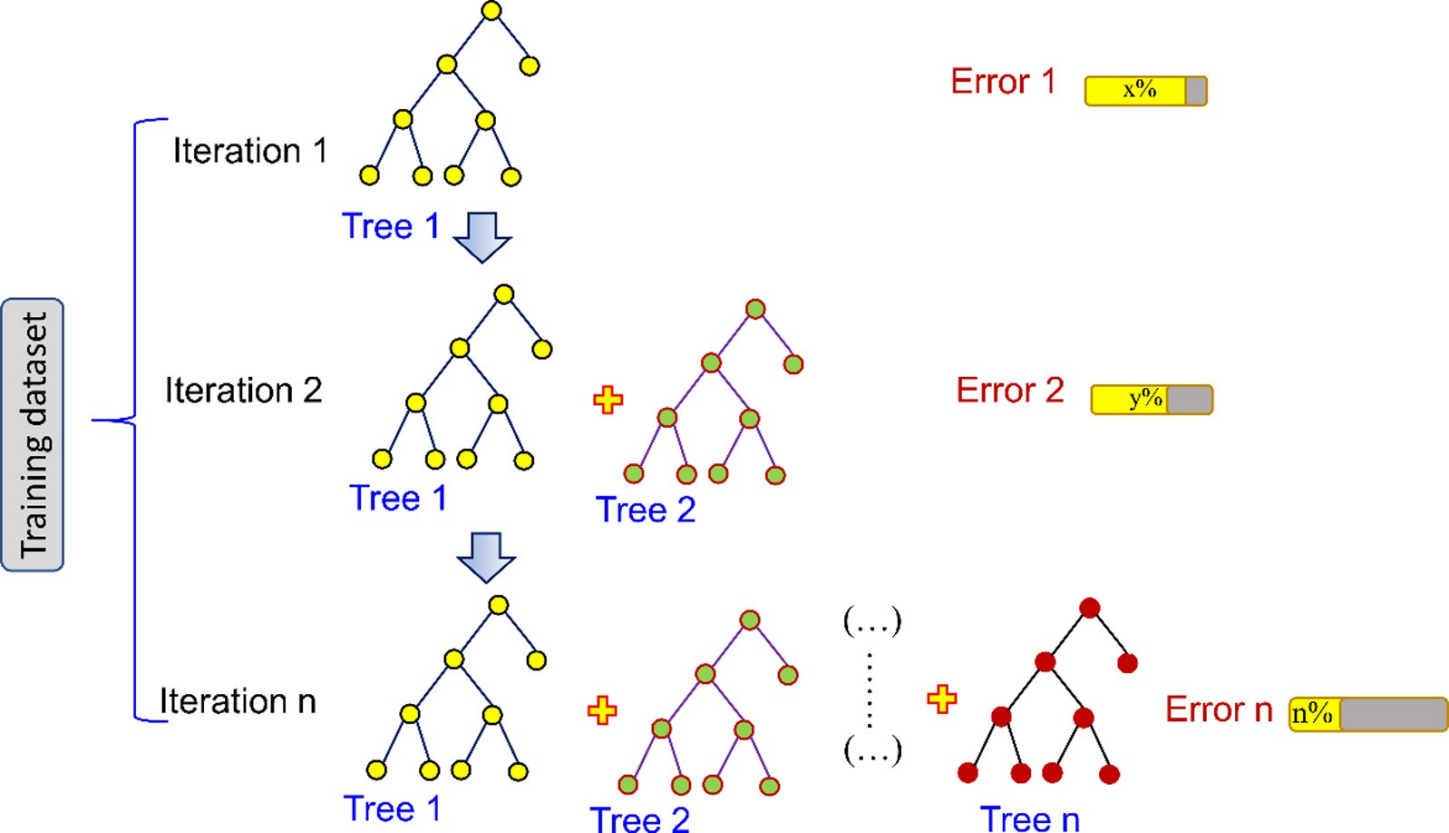
The framework of the GB algorithm.

Gradient boosting algorithm (GB), similar to the random forest, which is known as an ensemble technique firstly suggested by Friedman [40]. The fundamentals of the GB algorithm are on the basis of the boosting technique. The GB algorithm is established by combining the weak learner into a stronger learner in an iterative sequence [41]. The incorporation of different predictors from individual iterations could strengthen the performance of the model. In addition, in the GB algorithm, the total error and the loss of the model could be reduced [42]. As a result, the overfitting problem in the GB algorithm can be significantly decreased. In the GB technique, the weak learner is the regression tree, and in each iteration, the model was trained using stochastic gradient descent to lessen the error. The first weak learner (first tree) was learned in the GB algorithm to reduce the error in the first repetition. Then, the second tree (i.e. second weak leaner) was continuously trained and learned to minimize the error in the second iteration of the second tree. This process is conducted repeatedly until the desired error can be obtained. The framework of the GB algorithm is presented in **Fig 5**.

### 4.2. Evaluation of model performance

To evaluate the performance of the proposed models, different common indicators including determination coefficient (R^2^), root mean square error (RMSE), mean absolute error (MAE), and mean absolute percentage error (MAPE), were employed. The value of R^2^ varies from 0 to 1, the higher value of R^2^ indicates better performance of the model. In contrast, for three remaining indicators, the higher value of these indicators means the model has a low prediction, in contrast, the low values of these indicators indicate the model achieves high prediction accuracy. These indicators can be computed using the following equations:

$$R^{2}=\frac{\sum_{h=1}^{M}{\left(T_{h}^{act}-T_{avg}\right)}^{2}-\sum_{h=1}^{M}{\left(T_{h}^{act}-V_{h}^{est}\right)}^{2}}{\sum_{h=1}^{M}{\left(T_{k}^{act}-T_{avg}\right)}^{2}}$$ (1)

$$RMSE=\sqrt{\frac{1}{N}\overset{M}{\underset{h=1}{\Sigma }}{\left(T_{h}^{act}-V_{h}^{est}\right)}^{2}}$$ (2)

$$MAE=\frac{1}{N}\overset{M}{\underset{h=1}{\Sigma }}\left|V_{h}^{est}-T_{h}^{act}\right|$$ (3)

$$MAPE=\frac{1}{N}\overset{M}{\underset{h=1}{\Sigma }}\frac{\left|T_{h}^{act}-V_{h}^{est}\right|}{T_{h}^{act}}\times 100\%$$ (4)

where, $T_{h}^{act}$ and $V_{h}^{est}$are the experimental (actual) and estimated *h*^th^ value, respectively; N is the total number of samples in a dataset and $T_{avg}$is the mean of the experimental values.

## 4.3. K-Fold cross validation

**Fig 6:**
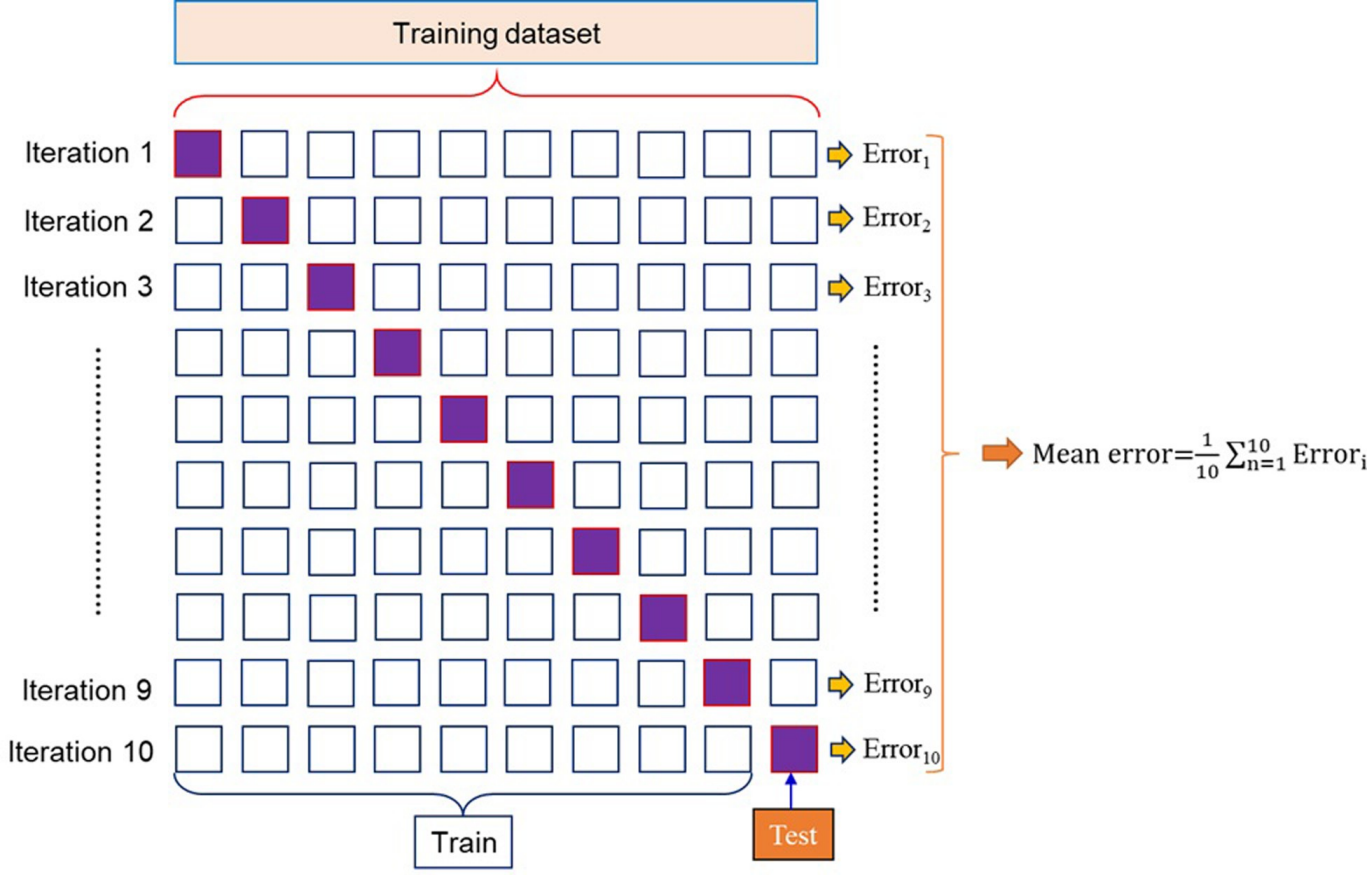
The structure of K-Fold validation process used in this study.

**Fig 7:**
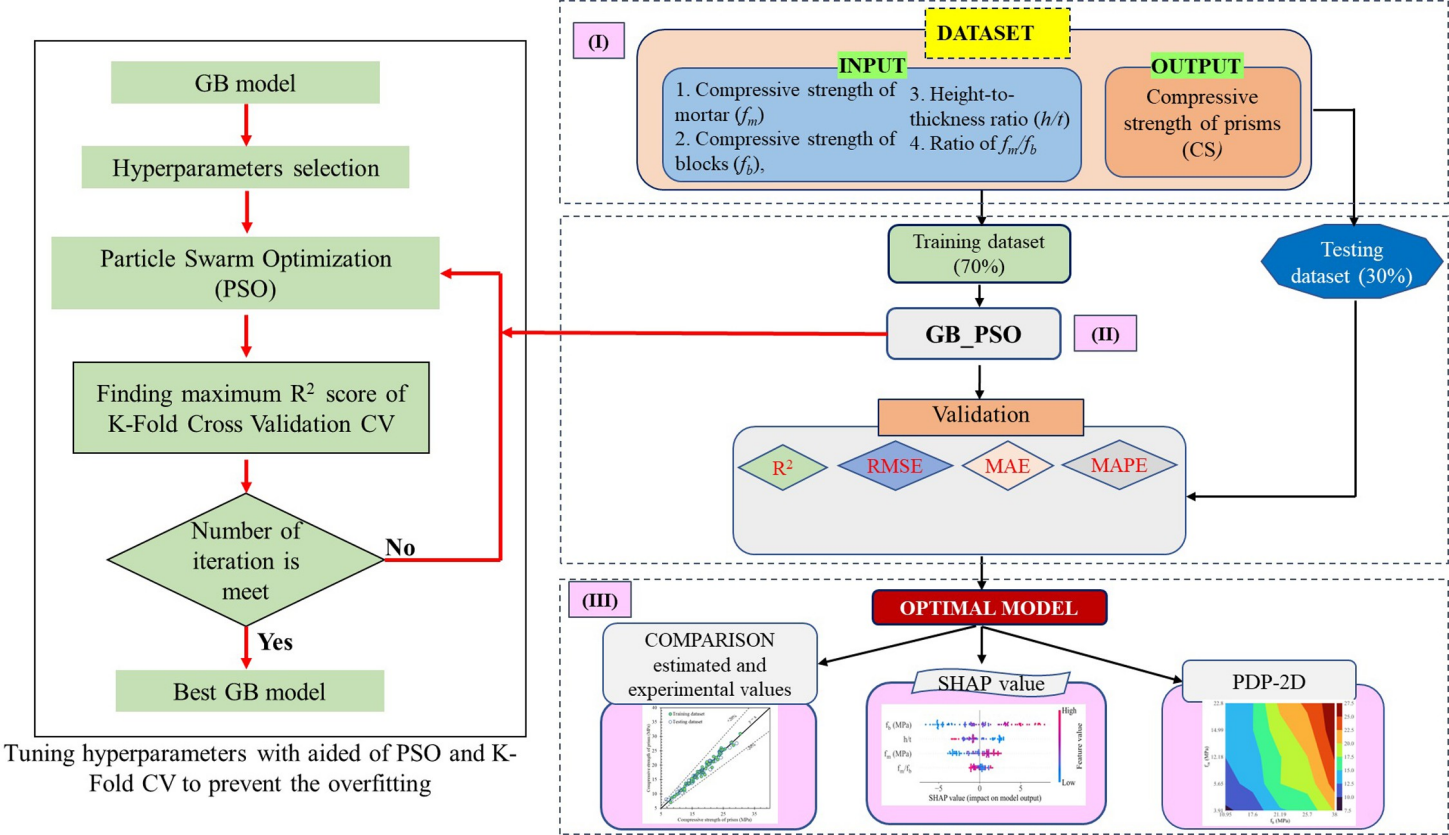
The flowchart used in the proposed in this study.

K-Fold Cross-Validation (CV) was employed to prevent the overfitting problem in this study [56–58]. The performance of the model was improved when the K-Fold cross-validation is used because the bias can be eliminated due to the random selection of training and testing from datasets. The original dataset was split into two parts, the first part consists of 70% of data is used for training, while the remaining part is used for testing. The K-Fold CV is only applied for the training dataset in this research. The training dataset was then divided into groups by K-Fold CV, one group for training and the remaining for testing. Through K-Fold cross-validation, 10 iterations were applied. For each iteration, by randomly divided the training dataset, the individual subset is generated, in which one subset is used for testing while 09 remaining subsets are used to train the model. The K-Fold CV was conducted 10 times to improve the performance of the model, and then the optimal model was achieved with the highest performance value such as maximal coefficient of determination R^2^, minimal Root Mean Square Error (RMSE) or minimal Mean Absolute Error (MAE). In fact, the cost function of tuning hyperparameters process (see later in Fig 7) is considered to be coefficient of determination R^2^ in this study. **Fig 6** shows the detail structure of K-Fold CV, the K-Fold CV technique with K = 10 is expressed as follows:

**Step 1:** The training dataset was split into ten subsets, in which 09 subsets are used for training model and one remaining subset is used for testing.
**Step 2:** In each iteration, the model was built based on the training subset then the model was verified using testing subset.
**Step 3:** The performance value of ML model as coefficient of determination R^2^ in each iteration and the mean value R^2^ of all subsets after reiterating were calculated.
**Step 4:** The model then was validated and based on the mean error after 10 times iteration, the optimal model was selected.

### 4.4. Methodology flowchart

**Fig 7** presents the flowchart of the GB model for estimating the CS of the masonry prism. In general, the flowchart consists of three major steps.

**Step 1:** Dataset preparation.

Entire input variables consisting of the compressive strength of mortar (f_m_), the compressive strength of blocks (f_b_), height-to-thickness ratio (h/t), and ratio of f_m_/f_b_ were prepared to create the machine learning model in this step. The number of the dataset is 102 hollow masonry concrete prisms, which were taken from the literature. After that, the dataset was divided into 2 categories, the first category (70% dataset) was used for training, while the remaining dataset (30% dataset) was used for validating the performance of the proposed model.

**Step 2:** Training and validating the model.

In this step, the proposed model was trained using a training dataset. In the training process, the GB algorithm was used to train the dataset. The hyperparameters of the GB model were obtained using Particle swarm optimization (PSO) considering coefficient of determination R^2^ score. The highest score R^2^ of tuning hyperparameters process was computed using K-Fold Cross-Validation with K = 10 iterations to prevent the overfitting issue.

In this step, the accuracy of the model was assessed via four indicators, including, R^2^, MAE, RMSE, and MAPE for testing dataset.

**Step 3:** Evaluating the feature’s importance

The optimal model was achieved based on the results of evaluation and validation in step 2. The SHAP was used to evaluate the importance of each input variable on the compressive strength of the hollow masonry prism. In addition, the importance of each and coupled input variable on the prediction accuracy of the output (the CS) was implemented using partial dependence plot 2D (PDP-2D). Finally, the estimated and actual values of the CS were compared to know the accuracy of the proposed model.

## 5. Results and discussion

### 5.1. Model hyperparamter tuning

**Fig 8:**
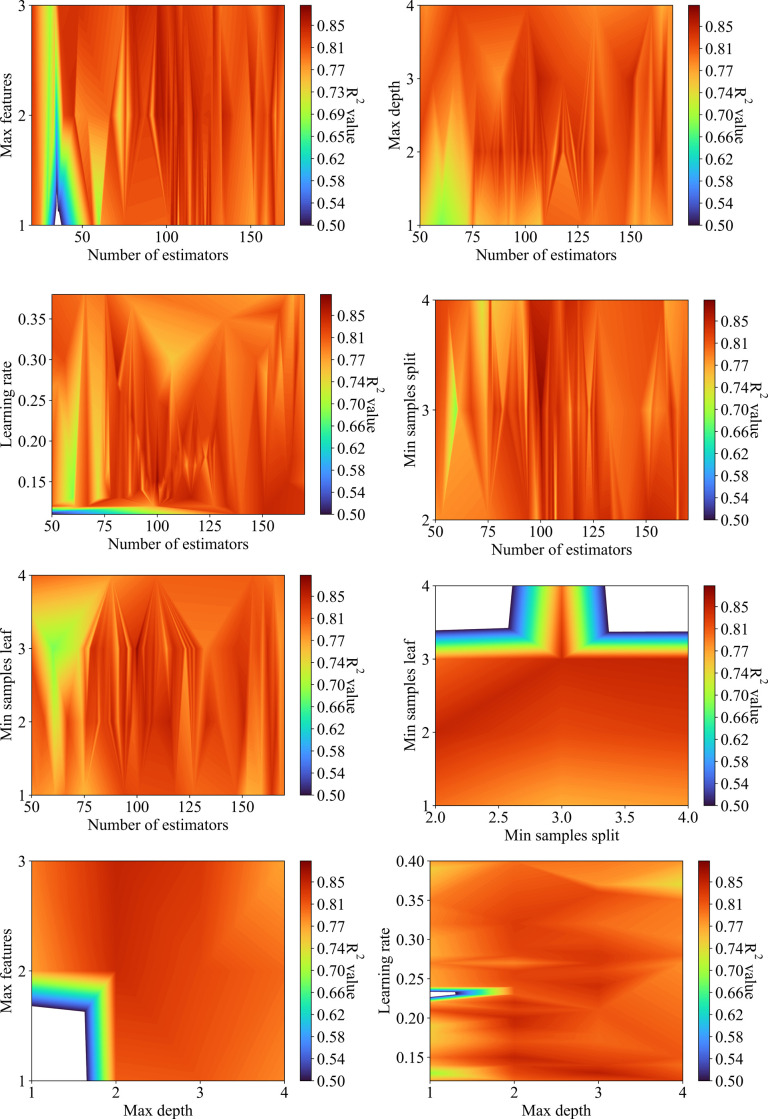
Values of R^2^ values with different hyperparameter values of the GB model.

To achieve the optimum hyperparameter of the proposed model, the Particle swarm optimization (PSO) was employed, and the performance index in the tuning process was estimated using R^2^ indicator. The hyper-parameter space of the proposed GB model used for tunning is presented in **Table 2**. **Fig 8** shows the surface plot of the R^2^ values with different hyperparameter values of the GB model. As shown in the figure, optimum parameter values can be obtained in all cases with a wide range. As shown in **Fig 8C**, the R^2^ values are higher than 0.85 and can be obtained when the learning rate is greater than 0.15 and the number of estimators is larger than 100 iterations. It can be observed that a max feature of 2 and a max depth of 2 resulted in the best performance of the model as presented in **Figs 8A**, 8B, 8G and 8H. The min samples split value of 3 and min samples leaf value of 3 led to the best results of R^2^, as shown in **Fig 8D**, 8E, and 8F. The optimum hyperparameters were obtained from the optimization analysis using PSO and these optimum hyperparameters are listed in **Table 2**.

**Table 2:** Hyperparameter space and optimum hyperparameter used in the proposed GB model.

Hyperparameter space		Optimum hyper-parameters	
Number of estimators	1–180	Number of estimators	100
Learning rate	0.1–0.4	Learning rate	0.15
Max depth	1–5	Max depth	2
Max features	1–4	Max features	2
Min samples split	2–5	Min samples split	3
Min samples leaf	1–5	Min samples leaf	3
Performance index	R^2^	Performance index	0.8830

### 5.2. Prediction of the compressive strength of the prism using GB model

**Fig 9:**
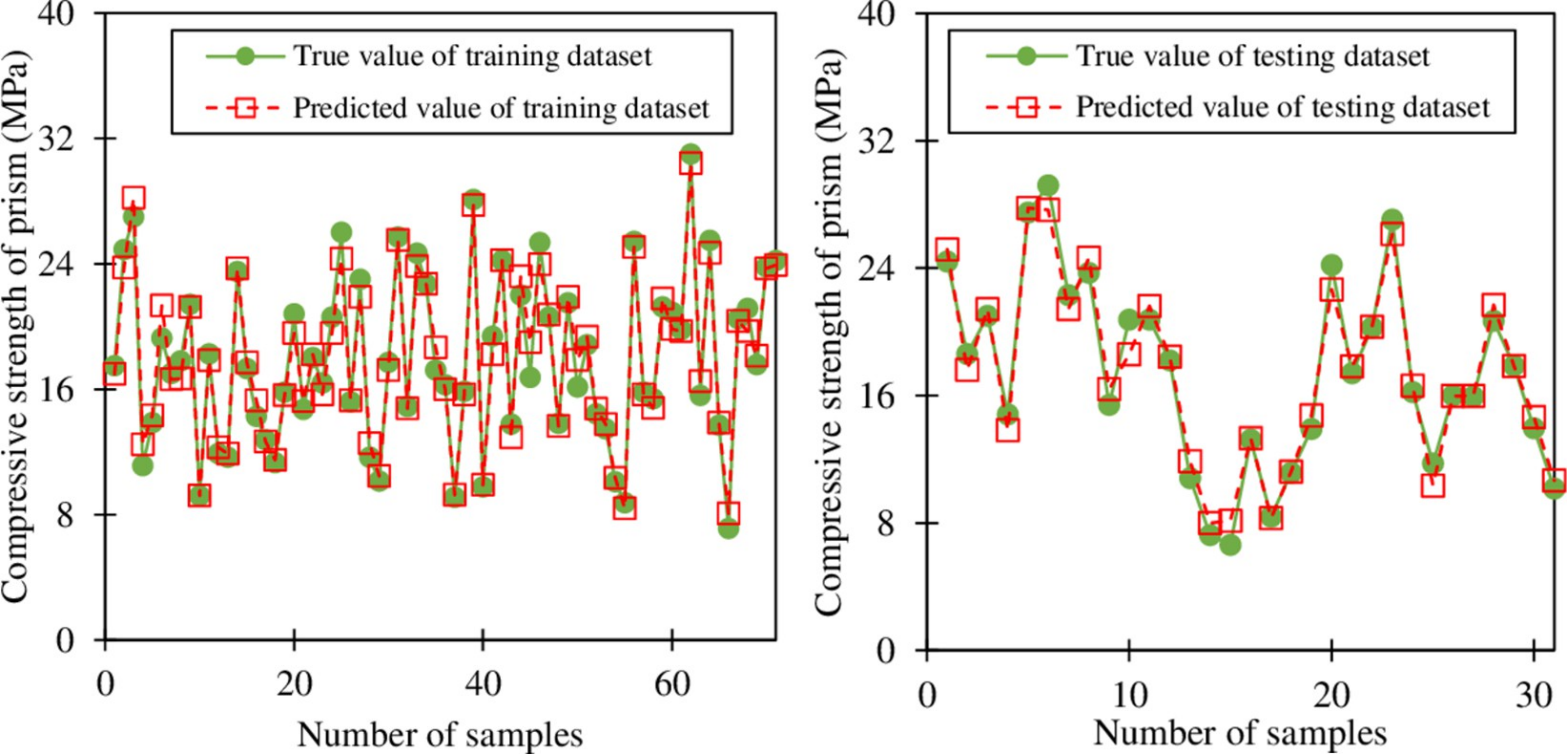
Comparison between actual and predicted outputs of the proposed GB model.

**Fig 9** presents the comparison between the predicted and actual results of the GB model for the compressive strength of the prism for both training and testing datasets. For the training dataset, the predicted values match well with the actual values, in which the values of R^2^, RMSE, MAE, and MAPE are **0.977**, 0.803 MPa, 0.612 MPa (minimal absolute error 0.011 MPa and maximal absolute error 2.243 MPa), and 0.036%, respectively. For the testing dataset, the predicted and actual values are almost the same, the values of R^2^, RMSE, MAE, and MAPE are **0.976**, 0.906 MPa, 0.728 MPa (minimal absolute error 0.002 MPa and maximal absolute error 2.177 MPa), and 0.048%, respectively. The results of R^2^ in this study are higher than those obtained from previous studies [20,29,38,59]. For example, the previous study performed by Fakharian et al. [38] used ANN, Gene Expression Programming (GEP), and Group Method of Data Handling (GMDH), to predict the CS of hollow concrete masonry blocks, the values of R^2^ range from 0.825 to 0.903. Besides, the previous study used six different machine learning models (decision tree, linear regression, random forest regression, ridge regression, ANN, and XGBoost), and the results indicated that the value of R^2^ ranges from 0.558 to 0.950. The better prediction accuracy of this study compared with previous studies can be due to the reduction of the overfitting problem. As aforementioned, the GB technique has a basic advantage in preventing overfitting problems and the K-Fold cross-validation and optimum hyperparameters were used in this study. This indicates that the proposed GB model used in the present study yielded a higher prediction accuracy in comparison with results obtained in previous works.

**Fig 10:**
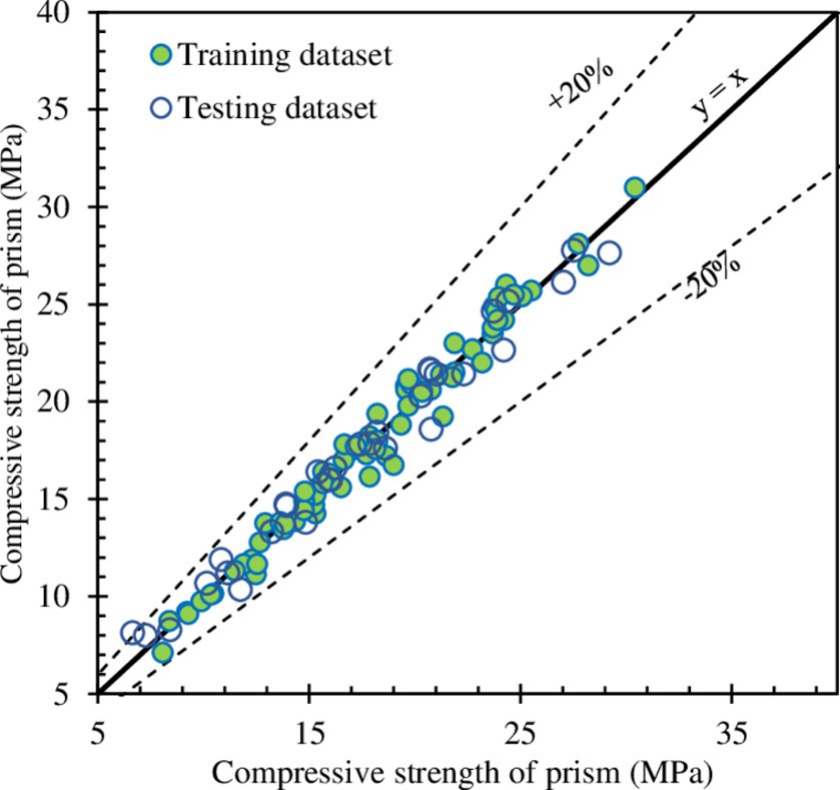
Comparison between the predicted and actual compressive strength.

The comparison between the predicted compressive strength and actual compressive strength is shown in **Fig 10**. It can be observed that the predicted and actual values match together well. Almost data points are located in the range of 20% error, there is only one point of the testing dataset that lies on the line y = ±1.2 x. In the previous study that used ANN, GMDH, and GEP, the results showed that the predicted and experimental values did not match well [38]. The results of the previous study showed that both training and testing were located outside the range of 20% error. Furthermore, another previous study [29] used different methods including CSA S304.1–04 (Canadian Standards Association), Eurocode 6, ANN, ANFIS, and the method proposed by Sarhat et al. [59] to predict the compressive strength of the hollow masonry prims, the results showed large scatter values, which also locate out the line y = ±1.2 x. Furthermore, a previous study used different machine learning models namely linear regression, decision tree, random forest, Ridge regression, ANN, and XGBoost showed that all models had a large number of datasets located out of the 20% error envelope [20]. The percentage of datasets out of 20% error range varied from 29 to 72%. The result of this study indicates that the proposed model performed better than other models in the previous studies and the models in this study also achieved a high accuracy in predicting the compressive strength of the hollow masonry prism.

**Fig 11:**
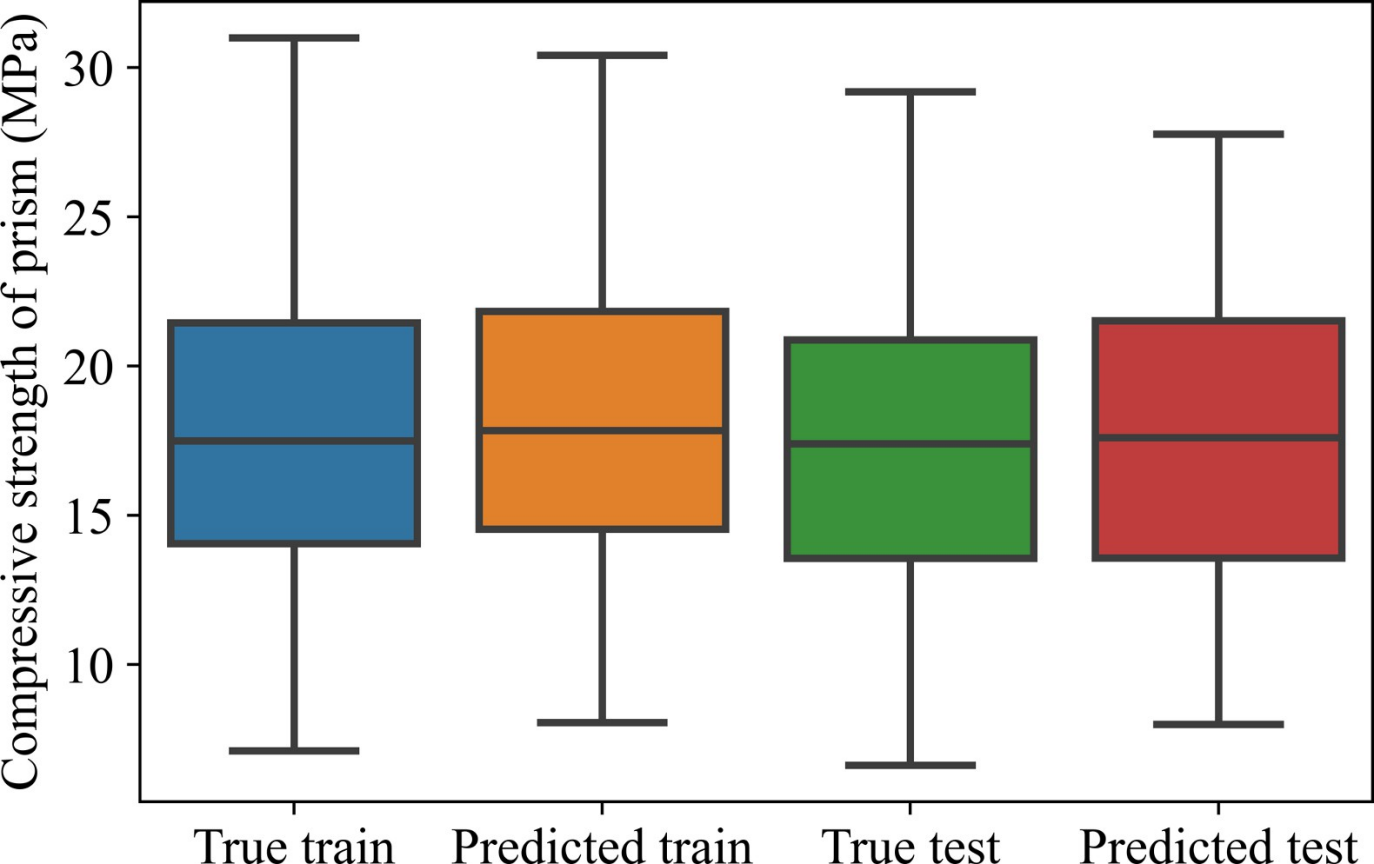
The values of compressive strength in training and testing.

**Fig 11** shows the values of the CS for both training and testing. From **Fig 11**, it can be seen that for both the train and test datasets, the predicted values have almost the same as the value of true values (actual values). In detail, for the training dataset, the predicted value is slightly higher than the actual value, however, the standard deviation of the predicted value is smaller than that of the actual value. Similar to the training dataset, for the testing dataset, the predicted values are also larger than the actual value, and the standard deviation of predicted values is also smaller than that of actual values.

Moreover, to ensure the usefulness the result of current manuscript, an excel calculation file including the complex equation generated from the GB model is also added in the revised manuscript (cf. S1 Data). This file facilitates a more streamlined process for engineers in designing compressive strength of hollow concrete masonry.

### 5.3. Feature importance analysis

**Fig 12:**
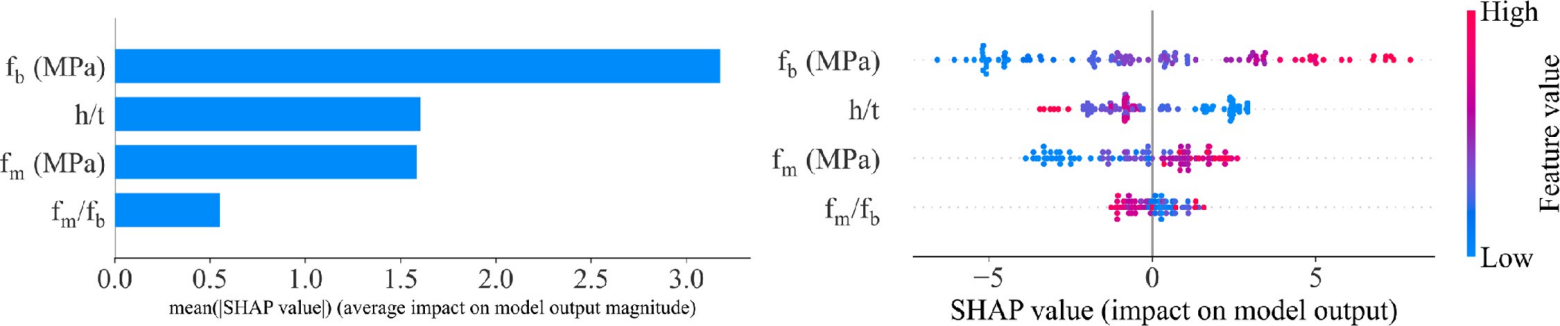
The SHAP values derived from the GB model.

The feature importance analysis of the CS of the prism is shown in **Fig 12**. The f_b_ is the most important variable influencing the CS of the hollow masonry prism. This is consistent with the results in previous works on predicting the CS of masonry prism using ML models [20,38]. The second vital factor is found for the h/t, and the f_m_ is considered as the third vital parameter. The least important variable is the f_m_/f_b_.

To understand the importance of each input variable, SHapley Additive exPlanation (SHAP) is used to calculate the contribution of each input variable [60,61]. The SHAP value is the mean marginal effect of individual variables via all possible combinations of input parameters. The importance of each input variable can be computed from the absolute SHAP value. The variables with a high absolute SHAP value are considered as important variables. The global feature importance is achieved by calculating the absolute SHAP value for all parameters, and then the absolute SHAP values are organized from big to small order. As shown in **Fig 12**, the x-axis shows the SHAP value and the y-axis presents the feature importance. The importance analysis of the CS of the hollow concrete specimens is presented in **Fig 12**. The point with red (or pink) indicates a high feature value corresponding to a higher value of SHAP. In addition, **Fig 12** shows a clear separation between positive and negative values of SHAP values. It can be observed that the most important variable that strongly affects the CS of hollow masonry prims is the f_b_ with a mean absolute SHAP value of 3.2. Because the f_b_ is the most important parameter of each hollow concrete block.

Regarding the f_b_ value, it can be observed that the higher value of f_b_ results in a greater SHAP value, and this result is consistent with the results obtained in previous studies for both experimental and modeling approaches [20,38]. The second crucial variable that strongly influences the CS of hollow masonry prisms is h/t with a mean absolute SHAP value of 1.63. From **Fig 12**, it can be seen that when the values of h/t increase, the lower SHAP value of h/t results in a higher value of feature importance. These results are also consistent with the results found in the previous work [20]. From these results of SHAP, it can be concluded that using SHAP could improve the understanding of the proposed model and also prove that the prediction accuracy of the proposed model is acceptable. As a result, it can be implied that SHAP can enhance the prediction ability of ML models.

**Fig 13:**
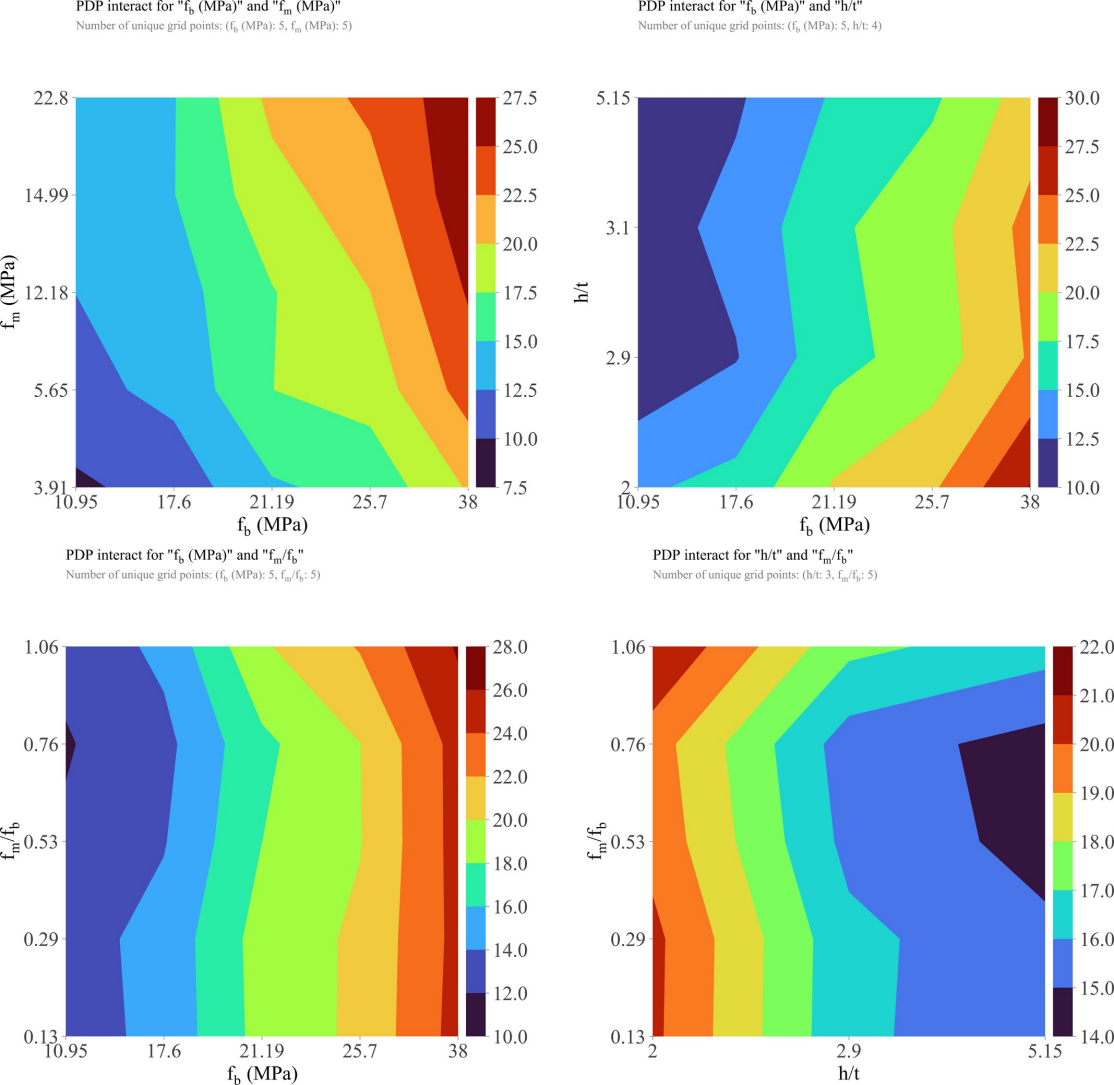
Partial dependence plots (PDP-2D) analysis of the coupled input factors affecting the CS of the hollow concrete specimens.

It is known that among input variables, the input variables have a mutual effect together, and these mutual influences greatly affect the CS of the hollow concrete prism. Thus, the partial dependence plot of two variables (PDP-2D) was shown to evaluate the influence of coupled variables on the compressive strength of the hollow concrete specimens as well as the mutual interaction between variables (**Fig 13**). It can be seen that with the value of f_b_ less than 21.19 MPa, the varies in f_m_ value have an insignificant influence on the compressive strength of the hollow concrete specimens (**Fig 13A**). However, when the values of f_b_ are greater than 21.19 MPa, the higher value of f_m_ significantly influences the CS of the hollow concrete specimens. This result is consistent with the results found in the previous studies [20]. **Fig 13B** shows that the values of h/t have little influence on the CS of the hollow concrete specimens regardless *f*_*b*_ values. The compressive strength slightly increases with the decrease in h/t value. Similarly, in the case of **Fig 13B**, the compressive strength of the hollow concrete specimens is almost independent of the value of f_m_/f_b_ with the change of f_b_ value. From **Fig 13**, a, b, c, it can be seen that the values of f_b_ greatly affect the CS of the hollow concrete specimens, the CS increases with the increasing f_b_ values. This result is in agreement with the results obtained in the previous studies [20,38]. These mutual effects of f_m_/f_b_ and h/t on the CS of the hollow concrete specimens are presented in **Fig 13D**. It can be observed that f_m_/f_b_ has a small influence on the CS with the change of h/t; while h/t values affect the CS of the hollow concrete specimens. The smaller values of h/t have a higher influence on the CS of the hollow concrete specimens. Based on the results of the feature importance analysis, it can be summarized that the f_b_ is the most variable that influences the CS of the hollow masonry concrete block.

## 6. Conclusions

This study evaluated and predicted the compressive strength (CS) of the hollow masonry prism using the Gradient Boosting model. 102 datasets taken from the literature were used for modeling. The input variables include the compressive strength of mortar (f_m_), the compressive strength of blocks (f_b_), the height-to-thickness ratio (h/t), the ratio of f_m_/f_b_, whereas, the output is the CS of the hollow concrete masonry block. The optimum hyperparameters of the model were obtained using PSO. The importances of individual and coupled input variables on the CS of the prims were evaluated using SHAP and PDP-2D.

- The GB model performed very well in evaluating and predicting the CS of the hollow masonry concrete blocks with a high prediction accuracy, the values of R^2^, RMSE, MAE, and MAPE are 0.977, 0.803 MPa, 0.612 MPa, and 0.036%, respectively.
- The prediction accuracy of the proposed GB model in this research is higher than other different machine learning models (such as decision tree, linear regression, random forest regression, ridge regression, ANN, and XGBoost) used in previous studies.
- The results of sensitivity analysis using SHAP and PDP-2D indicate that the compressive strength of blocks (f_b_) is the most dominant factor affecting the CS of the blocks. The second variable that strongly influences the CS of the prism is h/t ratio, while f_m_/f_b_ is the least important variable affecting the CS of the prism.
- From the result of this study, it can be concluded the proposed GB model provides a good method to evaluate and predict the CS of the hollow concrete masonry prism, which can bring valuable knowledge for design and practical application in this field.

The findings of this study indicated that the GB model has a good technique for predicting the compressive strength of the hollow masonry prims, which could support the design of the engineer in the practical application. However, the data employed in this study was sourced exclusively from several references with a medium data size. Future research should incorporate data from a broader range of sources to enhance the accuracy and generalizability of the predictive models.

## Supporting information

S1 Data (XLSX)
